# Challenges in the prophylaxis of severe respiratory syncytial virus infections

**DOI:** 10.1016/j.jped.2025.04.003

**Published:** 2025-05-22

**Authors:** Dana C. Feitosa, Sandra E. Vieira

**Affiliations:** a Faculdade de Medicina da Universidade de São Paulo, São Paulo, SP, Brazil; b Faculdade de Medicina da Universidade de São Paulo, Departamento de Pediatria, São Paulo, SP, Brazil

**Keywords:** Respiratory syncytial virus infections, Palivizumab, Medication adherence, Immunization, Passive, Infant, Premature

## Abstract

**Objective:**

To analyze palivizumab prophylaxis adherence among newborns and infants, as well as identify its challenges and facilitators.

**Methods:**

This retrospective study reviewed medical records of individuals who received palivizumab between 2008 and 2019 at a referral center in a metropolitan city in Brazil. Three adherence criteria were evaluated: an adequate number of doses received, interval between doses ≤ 35 days, and complete adherence (meeting both prior criteria). Associations between these criteria and sociodemographic/clinical variables, as well as post-prophylaxis bronchiolitis-related hospitalizations, were examined.

**Results:**

A total of 908 participants (mean age 6.7 months,50.8 % male,57.8 % residing in the city) were analyzed. During the three-season study period, a total of 1,158 doses were prescribed, and complete adherence was observed in 44.5 % of cases. Based on both the adequate number of doses and complete adherence criteria, lower adherence was noted among those living outside the city (52.8 % vs.60.9 %, *p* = 0.01; and 41.5 %vs.48.5 %, *p* = 0.03, respectively) and infants born to mothers younger than 20 years (39.7 % vs.60.3 %,*p* < 0.01; and 31.5 %vs.68.5 %, *p* = 0.02, respectively). Infants with gestational age < 28 weeks (65.8 % vs.34.2 %, *p* = 0.03) and birth weight < 1000 g (67.8 % vs.32.2 %, *p* = 0.03) had higher adherence under the adequate number of doses criterion. No association emerged between adherence and bronchiolitis-related hospitalizations, which were predominantly linked to maternal smoking during pregnancy and chronic lung disease.

**Conclusion:**

Adherence to palivizumab prophylaxis was low, highlighting the need to address geographic barriers and maternal age factors. Extreme prematurity and very low birth weight facilitated adherence, indicating that more targeted strategies or decentralized administration may improve outcomes in high-risk populations.

## Introduction

Respiratory syncytial virus (RSV) infection is one of the leading causes of hospitalization among infants. The most common clinical manifestation of severe infection is acute viral bronchiolitis, up to 90 % of bronchiolitis cases are attributed to RSV, especially in younger populations.^1^^,^^2^ In Brazil, the burden of RSV infection is evident from the approximately 180,000 hospital admissions of children under five years of age due to bronchiolitis/acute bronchitis, reflecting a significant impact on the healthcare system and emphasizing the need for effective preventive measures.^3^

The management of acute viral bronchiolitis is routinely based on supportive interventions.^1^^,^^2^ However, advances in medical technology have enabled the development of novel pharmacological prevention strategies.^4^, ^5^, ^6^, ^7^ Palivizumab, a humanized monoclonal antibody, is made available in Brazil through the Unified Health System (SUS) for specific high-risk populations vulnerable to severe illness and death. Access to Palivizumab has been regulated by the São Paulo State government since 2007.^8^ This monoclonal antibody protects against severe RSV infection by reducing hospitalizations; however, its dosing regimen (monthly intramuscular doses throughout the RSV season) and cost limit its widespread use.^4^

Several studies are currently underway to develop new prophylactic approaches for infants.^7^ Recently, a new monoclonal antibody, nirsevimab (Beyfortus, AstraZeneca, Cambridge, UK), was approved in the United States, Europe, and Brazil for single-dose intramuscular administration; however, it is not yet available through the Brazilian Unified Health System, leaving Palivizumab as the only form of passive immunization in the country.^5^^,^^6^ Additional vaccines have also been developed but are not yet included in the National Immunization Program. The maternal vaccine provides newborns with protection that must be complemented by monoclonal antibody administration starting in the first six months of life.^9^

Understanding the practical aspects of adherence to RSV prophylaxis, particularly the factors that hinder or contribute to its effectiveness is crucial. This knowledge will not only help improve the use of Palivizumab but also inform the implementation of new prevention strategies to be developed and offered by the Brazilian Unified Health System, such as potential vaccines and other products for infants requiring repeated doses.

## Methods

A retrospective study was conducted based on data collection from the computerized system of the Child and Adolescent Institute (ICr) and the Heart Institute (InCor) of the Faculty of Medicine of the University of São Paulo. Newborns and infants who received Palivizumab between 2008 and 2019 at the Special Vaccines and Immunobiological Unit of ICr (UVIE-ICr) were included. In this service, the administration of Palivizumab prophylaxis began in 2008 and follows the protocol established by the São Paulo State Health Department, which recommends prophylaxis for: infants under 1 year of age born at 28 weeks and 6 days or less of gestational age without other comorbidities (PT); preterm infants with chronic lung disease of prematurity requiring treatment in the last 6 months (CLD); and children with congenital heart disease under 2 years of age (CHD). Intramuscular doses of 15 mg/kg are administered during the RSV season in São Paulo, with an approximate interval of 30 days between doses.

Of the 4,655 indications for Palivizumab attended at UVIE-ICr during the study period, all individuals who received at least one dose of Palivizumab and were followed up at ICr or InCor were included. No exclusion criteria were applied.

Three criteria were adopted to assess adherence: the adequacy of the number of doses, considered adequate when the doses received matched 100 % of the prescribed doses; adequacy of the interval between doses, considered adequate when intervals were ≤35 days; and complete adherence when both the number of doses and the intervals were adequate.

Sociodemographic and clinical characteristics, as well as clinical outcomes (hospitalizations and deaths), of the participants were described. Analyses of factors influencing adherence were performed based on sociodemographic and clinical characteristics, the RSV circulation season experienced by the child, and the criteria for prophylaxis indication. Associations between adherence rates and hospitalizations due to bronchiolitis after the start of prophylaxis were also analyzed.

### Ethical considerations

The study was conducted in compliance with the principles outlined in the Declaration of Helsinki and was approved by the Ethics Committee of the Faculty of Medicine of the University of São Paulo (approval number 3.974.703). All measures were taken to ensure the confidentiality and privacy of the participant's data.

### Statistical analysis

A convenience sample was used in this study. Considering the variable “maternal age” and the outcome “complete adherence,” the estimated power to reject the null hypothesis was 80 %, adopting a 95 % confidence interval. Categorical variables were compared using the chi-square test and Fisher's exact test, while continuous variables were compared using the paired Student's *t*-test. Logistic regression analyses were performed to examine associations among variables potentially related to the outcomes. A 95 % confidence interval and *p*-values < 0.05 were considered significant. SPSS 20 software was used for the analysis.^10^

## Results

A total of 916 infants were eligible, but eight medical records were lost, leaving 908 infants for analysis. During the second RSV season, 240 infants received prophylaxis, and nine received it during the third RSV season.

### Sociodemographic and clinical characteristics

White ethnicity, male sex, and residence in the city of São Paulo (SP) were predominant. At birth, the mean weight was 1,932 g (range: 440–4,600 g), and the mean gestational age was 33 weeks and 2 days. The mean maternal age was 29.9 years (± 7.11). At the time of the first dose, the participant's age ranged from 0 to 25 months and weight ranged from 660 g to 13,875 g. The sociodemographic and clinical characteristics are shown in Table 1.

**Table 1:** Sociodemographic and clinical characteristics of 908 infants who received Palivizumab between 2008 and 2019.

Characteristics of infants	*n* (%)
Sex	
Female	432 (47.6)
Male	462 (50.9)
Ethnicity	
White	479 (52.8)
Black	30 (3.3)
Asian	2 (0.2)
Mixed-race	149 (16.4)
Age (months)	
Mean age at first Palivizumab dose	6.7 (±5.3)
Weight (grams)	
Mean weight at first Palivizumab dose	5355 (±2.5)
Place of residence	
City of São Paulo	525 (57.8)
Other cities	368 (40.5)
Maternal age (years)	
<20	65 (7.2)
21–30	245 (27.0)
>30	277 (30.5)
Maternal smoking during pregnancy	
Smokers	48 (5.3)
Non-smokers	329 (36.2)
Indications for Palivizumab	
GA^a^ <29 weeks without chronic lung disease	100 (11.0)
Chronic lung disease of prematurity (CLD)	326 (35.9)
Congenital heart disease (CHD)	458 (50.4)
First palivizumab dose	
Administered in the maternity ward	179 (19.7)
On oxygen therapy^b^	101 (14.0)
Deaths during the study period	
CHD	14 (1.5)
CLD	5 (0.6)
Total deaths	19 (2.1)

a GA, gestational age.

b Received home oxygen therapy at the time of the first dose.

### Analyses of adherence to palivizumab prophylaxis

During the three-season study period, a total of 1,158 doses were prescribed. Among participants, 650 (56.2 %) received the recommended number of doses, 763 (75.2 %) adhered to the recommended intervals between doses, and 515 (44.5 %) achieved full adherence.

No differences in adherence were found with respect to sex, ethnicity, or maternal smoking during pregnancy. An inadequate number of doses and lack of full adherence were more frequent in younger mothers. Infants born at <29 weeks’ gestational age showed higher adherence, according to the “adequate number of doses” criterion, when compared to those with CHD and CLD, as did those with a birth weight < 1000 g. Participants from São Paulo showed higher adherence than those from other cities, according to both the “adequate number of doses” and the “full adherence” criteria (Table 2).

**Table 2:** Associations between adherence to prophylaxis and the characteristics of infants who received Palivizumab between 2008 and 2019.

	Adherence criterion								
	Number of doses^a^			Interval between doses^b^			Full adherence^c^		
Characteristics	Inadequate	Adequate	*p*^e^	Inadequate	Adequate	*p*^e^	No	Yes	*p*^e^
Sex									
Female	176 (45.7 %)	260 (49.7 %)	0,23	98 (49.2 %)	280 (47.5 %)	0,66	227 (46.0 %)	209 (50.5 %)	0.17
Male	209 (54.3 %)	263 (50.3 %)		101 (50.8 %)	310 (52.5 %)		267 (54.0 %)	205 (49.5 %)	
Ethnicity									
White	209 (74.4 %)	273 (71.5 %)	0.44	106 (70.7 %)	315 (73.4 %)	0.67	263 (72.3 %)	219 (73.2 %)	0.16
Black	10 (3.6 %)	20 (5.2 %)	0.29	6 (4.0 %)	19 (4.4 %)	0.82	13 (3.6 %)	17 (5.7 %)	0.19
Asian	0 (0.0 %)	2 (0.5 %)	0.22	0 (0.0 %)	2 (0.5 %)	0.40	0 (0.0 %)	2 (0.7 %)	0.12
Mixed-race	62 (22.1 %)	87 (22.8 %)	0.92	38 (25.3 %)	93 (21.7 %)	0.36	88 (24.2 %)	61 (20.4 %)	0.25
Maternal smoking during pregnancy									
Yes	20 (14.5 %)	28 (11.7 %)	0,43	7 (7.6 %)	34 (14.0 %)	0,10	23 (12.0 %)	25 (13.4 %)	0.68
No	118 (85.5 %)	211 (88.3 %)		85 (92.4 %)	208 (86.0 %)		168 (88.0 %)	161 (86.6 %)	
Maternal age (years)									
<20	44 (14,6 %)	29 (6,5 %)	<0.01	15 (8,7 %)	42 (8,6 %)	0,90	50 (12,6 %)	23 (6,6 %)	0,02
21–30	123 (40,9 %)	188 (42,4 %)	0.06	73 (42,2 %)	197 (40,4 %)	0.67	164 (41,2 %)	148 (42,7 %)	0.69
>30	134 (44,5 %)	226 (51,0 %)	0.08	85 (49,1 %)	249 (51,0 %)	0.67	184 (46,2 %)	176 (50,7 %)	0.22
Prophylaxis indication^d^									
AG <29 weeks	31 (31.0 %)	69 (69.0 %)	0.02	27 (29.7 %)	64 (70.3 %)	0.58	50 (50.0 %)	50 (50.0 %)	0.42
DPC	131 (40.2 %)	195 (59.8 %)		66 (23.2 %)	218 (76.8 %)		170 (52.1 %)	156 (47.9 %)	
CC	210 (45.9 %)	248 (54.1 %)		105 (26.3 %)	295 (73.8 %)		261 (57.0 %)	197 (43.0 %)	
Birth weight									
<1,000 g	65 (19.9 %)	137 (28.7 %)	0.03	47 (26.4 %)	135 (25.6 %)	0.67	95 (22.2 %)	107 (28.5 %)	0,14
1,000−1,499 g	71 (21.8 %)	103 (21.6 %)	0.95	34 (19.1 %)	119 (22.5 %)	0.33	92 (21.5 %)	82 (21.9 %)	0.90
1,500–2,499 g	66 (20.2 %)	76 (15.9 %)	0.11	35 (19.7 %)	87 (16.5 %)	0.34	84 (19.6 %)	58 (15.5 %)	0.12
≥2,500 *g*	124 (38.0 %)	161 (33.8 %)	0.21	62 (34.8 %)	187 (35.4 %)	0.89	157 (36.7 %)	128 (34.1 %)	0.45
Gestational age (weeks)									
<29	55 (16.2 %)	106 (21.6 %)	0,03	39 (21.2 %)	108 (19.9 %)	0,52	80 (18.1 %)	81 (20.9 %)	0,26
29–31	83 (24.4 %)	136 (27.8 %)	0,28	45 (24.5 %)	147 (27.1 %)	0,47	110 (24.8 %)	109 (28.2 %)	0,27
32–33	16 (4.7)	30 (6.1 %)	0,38	10 (5.4 %)	33 (6.1 %)	0,74	24 (5.4 %)	22 (5.7 %)	0,87
34–36	47 (13.8 %)	49 (10.0 %)	0,09	27 (14.7 %)	52 (9.6 %)	0,06	60 (13.5 %)	36 (9.3 %)	0,06
37–41	135 (39.7 %)	168 (34.3 %)	0,11	62 (33.7 %)	198 (36.5 %)	0,49	165 (37.2 %)	138 (35.7 %)	0,63
≥42	4 (1.2 %)	1 (0.2 %)	0.16	1 (0.5 %)	4 (0.7 %)	1,00	4 (0.9 %)	1 (0.3 %)	0,37
Place of residence									
City of São Paulo	208 (39.1 %)	324 (60.9 %)	<0.01	112 (23.9 %)	356 (77.7 %)	0.30	274 (51.5 %)	258 (48.5 %)	0.03
Other cities	177 (47.2 %)	198 (52.8 %)		87 (27.2 %)	233 (72.8 %)		220 (58.6 %)	155 (41.3 %)	

a Adequate number of doses means that the number of doses received was equal to the number of doses prescribed.

b Adequate interval means an interval between doses of less than or equal to 35 days.

c Full adherence means receiving all indicated doses and all intervals ≤35 days.

d Gestational age at birth; CLD, chronic lung disease of prematurity; CHD, congenital heart disease.

e Chi-square test or Fisher’s exact test when *n* < 5. For comparisons among more than one category, each category was compared to the sum of the others.

### Analysis of hospitalizations after initiation of prophylaxis

After prophylaxis began, 473 participants (52.1 %) were hospitalized for any cause (regardless of admission diagnosis). Of these, 174 (36.7 %) were hospitalized with a diagnosis of bronchiolitis, 281 (59.4 %) for other causes, and 18 (3.8 %) for unspecified causes.

No statistically significant differences were found in the association between bronchiolitis-related hospitalizations and any of the adherence criteria. Hospitalization for any cause was more frequent among those with lower adherence according to the “adequate number of doses” and “full adherence” criteria (Table 3).

**Table 3:** Associations between Palivizumab prophylaxis adherence criteria and admission diagnosis in infants hospitalized after prophylaxis initiation.

Admission diagnosis	Number of doses			Intervals between doses			Full adherence		
	Inadequate N (%)	Adequate N (%)	*p*-value	Inadequate N (%)	Adequate N (%)	*p*	No N (%)	Yes N (%)	*p*-value
Bronchiolitis *N* = 174	90 (41.7)	84 (35.1)	0.15	34 (30.9)	117 (41.1)	0.06	106 (39.4)	68 (36.6)	0.53
Any diagnosis *N* = 473	228 (74.8)	245 (57.6)	<0.01	114 (66.7)	297 (63.1)	0,40	282 (70.5)	191(57.9)	<0.01

No associations were found between sex, ethnicity, or place of residence and bronchiolitis hospitalization. Bronchiolitis-related hospitalizations were more frequent among infants whose mothers smoked during pregnancy, infants with CLD, lower gestational age, lower birth weight, use of medications for chronic lung disease of prematurity, and oxygen therapy. Hospitalizations for other causes were more frequent among those with congenital heart disease (Table 4).

**Table 4:** Associations between clinical characteristics and hospital admission diagnosis in infants hospitalized after prophylaxis initiation.

	Diagnosis		
Characteristics of hospitalized infants	Bronchiolitis *N* = 174	Other diagnosis *N* = 274	*p*-value
Birth weight (grams)			
<1,000	46 (27.7 %)	44 (16.7 %)	<0.01
1,000–1,499	38 (22.9 %)	39 (14.8 %)	0.03
1,500–2,499	27 (16.3 %)	47 (17.9 %)	0.07
2,500	55 (33.1 %)	133 (50.6 %)	<0.01
Gestational age (weeks)			
<28	42 (24.9 %)	35 (13.1 %)	<0.01
28–31	44 (26.0 %)	43 (16.1 %)	0.01
32–33	7 (4.1 %)	14 (5.2 %)	0.60
34–36	18 (10.7 %)	43 (16.1 %)	0.11
37–41	58 (34.3 %)	129 (48.3 %)	<0.01
≥42	0 (0.0 %)	3 (1.1 %)	0.28
Prophylaxis indication^a^			
GA <29 weeks without CLD	19 (10.9 %)	22 (7.9 %)	0.30
CLD	75 (43.1 %)	66 (23.6 %)	<0.01
CHD	80 (46.0 %)	186 (66.4 %)	<0.01
Therapies in use at the time of Palivizumab administration			
Bronchodilator	27 (16.5 %)	18 (7.3 %)	<0.01
Inhaled corticosteroid	54 (32.9 %)	40 (16.1 %)	<0.01
Oxygen therapy	30 (18.2 %)	27 (9.9 %)	0,01
Sex			
Female	79 (45.4 %)	127 (45.2 %)	0.97
Male	95 (54.6 %)	154 (54.8 %)	
Place of residence			
City of São Paulo	97 (55.7 %)	172 (61.2 %)	0.25
Other cities	77 (44.3 %)	109 (38.8 %)	
Maternal smoking during pregnancy	15 (17.4 %)	8 (8.1 %)	0.05

a GA, gestational age at birth; CLD, chronic lung disease of prematurity; CHD, congenital heart disease.

Three multivariate models were constructed to evaluate factors potentially associated with bronchiolitis-related hospitalization. Each model considered a different adherence outcome: Model 1 – adequate number of doses; Model 2 – adequate interval between doses; Model 3 – full adherence. The likelihood of bronchiolitis-related hospitalization was higher in infants with chronic lung disease of prematurity (Table 5).

**Table 5:** Multivariate analyses of variables potentially associated with bronchiolitis-related hospitalization after prophylaxis initiation, according to the adherence criteria.

Model (according to adherence criterion)	OR^a^	IC 95 %^b^		*p*-value
Model 1 - adequate number of doses^e^				
Adequate number of doses	0,79	0,46	1,36	0,40
IG^c^ ≤9 weeks	1,81	0,79	4,11	0,16
CLD^d^	4,42	2,28	8,59	0,00
Congenital heart disease	1,01	0,80	1,25	1,00
Maternal smoking during pregnancy	0,52	0,22	1,20	0,13
Use of oxygen after discharge	0,91	0,44	1,88	0,80
Residence in São Paulo	0,99	0,56	1,75	0,97
Model 2 - adequate interval between doses^f^				
Adequate intervals between doses	1,58	0,84	2,96	0,16
IG^c^ ≤29 weeks	2,08	0,85	5,08	0,11
CLD^d^	4,09	1,97	8,48	0,00
Congenital heart disease	1,01	0,73	1,43	1,00
Maternal smoking during pregnancy	0,41	0,17	1,02	0,06
Use of oxygen after discharge	0,82	0,37	1,79	0,61
Residence in São Paulo	0,95	0,51	1,76	0,87
Model 3 - full adherence g				
Full Adherence	0,94	0,55	1,61	0,83
IG^c^ ≤29 weeks	1,81	0,79	4,11	0,16
CLD^d^	4,49	2,31	8,74	0,00
Congenital heart disease	1,01	0,73	1,43	1,00
Maternal smoking during pregnancy	0,50	0,22	1,18	0,11
Use of oxygen after discharge	0,92	0,45	1,90	0,83
Residence in São Paulo	1,00	0,56	1,76	0,99

a OR, odds ratio.

b 95 % CI, 95 % confidence interval.

c GA, gestational age at birth.

d CLD, chronic lung disease of prematurity.

e Adequate number of doses, number of doses received equals number of doses indicated.

f Adequate interval, interval between doses ≤35 days.

g Full adherence, receiving all indicated doses and all intervals ≤35 days.

## Discussion

The retrospective analysis of a 12-year period showed low adherence to prophylaxis against severe RSV infection with Palivizumab. Living outside the city where the drug was administered and lower maternal age compromised adherence, whereas low birth weight contributed to higher adherence rates.

The study carried out in the city of São Paulo highlighted the difficulty imposed by the distance between participants’ residences and the healthcare service where Palivizumab was administered. São Paulo is a large metropolitan city with heavy vehicle traffic. Because it is a high-cost medication, the monoclonal antibody is offered free of charge by the government and is available in only a few referral centers, meaning that those residing in more distant areas may face obstacles to accessing prophylaxis. Monthly administration during the viral season requires up to five visits to the health service. In the literature, some studies report that traveling long distances and lack of transportation compromise adherence.^11^^,^^12^ In countries such as the United States, Canada, and France, where administration is available at home or in clinics close to patients’ homes, adherence rates reach 80–95 %.^12^, ^13^, ^14^

In addition to distance, other sociodemographic aspects may interfere with adherence. The present findings emphasize lower adherence among infants whose mothers were younger, especially those under 20 years of age. This association has also been reported in previous studies.^11^^,^^15^ It is possible that older mothers may have a better understanding of prophylaxis, stronger family support, and higher education levels, all of which favor adherence. Several previous studies have shown that higher maternal education is associated with better adherence.^14^, ^15^, ^16^, ^17^

Interestingly, infants born at <29 weeks gestational age showed higher adherence, whereas those with congenital heart disease (CHD) or chronic lung disease of prematurity (CLD) were not associated with different adherence rates. Extremely preterm infants often remain hospitalized in the maternity ward for a longer period, facilitating the administration of the first doses in the neonate hospital setting.^17^^,^^18^ Previous studies have reported that initiating prophylaxis in younger infants helps improve adherence.^14^^,^^17^^,^^19^^,^^20^ It is also possible that, due to the greater severity of their underlying diseases, those with CHD and CLD may have attended fewer administrations due to clinical instability or hospitalizations. In this study, the authors found that infants with CHD were hospitalized more frequently for any cause and accounted for the majority of deaths in the sample. Adherence associated with different prophylaxis indications remains controversial and may vary across different regions. Contrary to these results, the French EPIPAGE-2 study showed that children with more severe pulmonary conditions had higher adherence.^17^

In line with the finding that preterm infants demonstrated higher adherence, lower birth weight was also associated with better adherence. This may be related to the perception among families of increased vulnerability in these infants, as previously described.^14^^,^^17^

No sex- or ethnicity-related differences in adherence were observed. Other studies have shown lower adherence among male infants.^19^^,^^20^ According to these authors, although males have a higher risk of RSV-related hospitalization, their families may perceive them as more resilient and less in need of prophylaxis.^19^

Previous studies have also reported worse adherence among those of African descent compared to White individuals, likely due to socioeconomic conditions.^11^^,^^14^, ^15^, ^16^ It is possible that this discrepancy with these findings may be linked to free access to prophylaxis. Despite socioeconomic inequalities, the provision of Palivizumab by the Brazilian National Health System (SUS) may have minimized ethnic differences in adherence.^21^

Another important aspect involves the association between adherence criteria and clinically significant outcomes after prophylaxis initiation. Although it is well established that Palivizumab prophylaxis reduces hospitalizations due to RSV infections,^22^ in the present study the authors did not observe an association between treatment adherence and subsequent bronchiolitis-related hospitalizations. These hospitalizations were associated with well-known risk factors such as CLD, prematurity, maternal smoking during pregnancy, and factors related to the severity of the underlying diseases. Nonetheless, the lack of influence of treatment adherence on bronchiolitis-related hospitalizations may suggest that even incomplete prophylaxis conferred some degree of protection. On the other hand, among those who received an adequate number of doses and had full adherence, hospitalizations for any cause were less frequent. It is possible that some of these diagnoses included those associated with severe RSV infection, such as pneumonia and wheezing episodes. In the study by Chan et al.,^16^ adherence was also not associated with RSV hospitalizations, but better adherence was linked to lower rates of intubation, ICU admissions, and respiratory support. Whether increased adherence to prophylaxis reduces hospitalizations for RSV infections remains controversial in the literature. The lack of uniformity in adherence parameters complicates the analysis. A systematic review showed that the association between reduced hospitalizations and adherence to prophylaxis varied according to the adherence criteria adopted in each study.^14^

This study has some limitations inherent to its design. The absence of data on maternal education and the families’ socioeconomic status in medical records prevented statistical analysis of these variables, although the available analysis of maternal age is likely a proxy for education level.^14^, ^15^, ^16^, ^17^

Another limitation lies in the reasons for missed doses or delayed intervals. Actual dose administration depends on whether the health system indicates prophylaxis, the bureaucratic processes involved in authorizing the indicated doses, and access to the healthcare system, which can delay the start of prophylaxis each season. Many participants may also have missed doses or postponed intervals due to a lack of clinical stability to receive the doses, such as in the event of infections or hospital admissions for decompensation of their underlying disease.

The present findings, considering the sociodemographic characteristics of the participants and the influence of these factors on adherence, may contribute to improving the efficiency of preventing severe RSV infection. Given that Palivizumab is a high-cost medication and has an impact on the morbidity and mortality of the population for which it is indicated, such factors are even more relevant in developing countries like Brazil, where a significant portion of the population has low education and socioeconomic levels, and access to public health services may be decisive for healthcare.^21^

The adherence to Palivizumab prophylaxis found in this study was lower than that reported in most of the adherence studies in the literature.^11^^,^^13^^,^^15^^,^^17^ Coupled with this low adherence is the fact of living in a different city than the administration site, as well as lower adherence among participants who were indicated to receive the maximum number of doses. These findings suggest the need for decentralization and regionalization of immunoprophylaxis administration, as well as reducing the number of health facility visits through the development of products with longer half-lives and fewer required doses.^12^

Currently, a new monoclonal antibody, nirsevimab, has been approved by the Brazilian Health Regulatory Agency and was previously approved in the United States and Europe.^23^, ^24^, ^25^ It has an extended half-life, allowing a single-dose regimen per RSV season, and is also effective for indications beyond those of Palivizumab.^5^ The low full-adherence rate to Palivizumab prophylaxis found in this study reinforces the importance of incorporating nirsevimab into the public health system for free distribution nationwide. However, since it is also indicated for all infants under 8 months of age born during or entering the RSV season, cost-effectiveness studies must be conducted. Currently, the Brazilian Society of Pediatrics recommends nirsevimab for all newborns and infants under one year of age who are born at the beginning of or during the RSV season. This monoclonal antibody is also recommended for infants with the same indications as Palivizumab, as well as other risk situations for severe RSV infection.^26^

Another recently available prophylactic tool is the RSV vaccine for pregnant women, which leads to the transplacental transfer of antibodies and protects infants against RSV infection in the first six months of life. This vaccine, which is currently available, has received authorization from Anvisa and can be administered to older adults and pregnant women in the second or third trimester as a single intramuscular dose.^9^

It is important to underscore that, beyond emphasizing the need for more effective prophylactic regimens against RSV infection, identifying and addressing barriers to adequate adherence can improve other immunization strategies and outpatient prophylactic treatments in infants who require repeated healthcare visits over extended periods.

In summary, the present findings highlight critical factors that hinder palivizumab adherence within the Brazilian Unified Health System. Offering other prophylactic options, such as new monoclonal antibodies and vaccines, alongside decentralized healthcare strategies, may help mitigate logistical and socioeconomic barriers, ultimately contributing to more effective RSV prevention in Brazil’s pediatric population. Until this measure is effectively implemented, it is crucial that health professionals, parents, and caregivers remain informed about the risk factors outlined herein to enhance adherence rates. The effectiveness of the immunization program largely depends on the efforts of personnel responsible for conducting telephone follow-ups, reminding patients of Palivizumab injection schedules, facilitating communication with local family physicians, and coordinating appointments. Conversely, the decline in adherence to dose administration is partly attributable to the limited availability of healthcare professionals in certain regions of the country. Regarding the administration of Palivizumab, several obstacles persist, among which anti-vaccine and bioecological trends represent a significant factor of resistance.^27^ It is essential to provide evidence-based information to caregivers through programmatic actions, in order to empower and support them in promoting the necessary protection for premature children.

## Declaration of generative AI in scientific writing

During the preparation of this work, the authors used Chat GPT in order to improve readability and the English language. After using this tool/service, the authors reviewed and edited the content as needed and took full responsibility for the content of the publication.

## Funding sources, or names of institutions or companies providing equipment and materials

Nothing to declare.

## Author's contribution

DCF: aquisition of data, analysis and interpretation of data, drafting the article, final approval. SEV: conception and design of the study, analysis and interpretation of data, revising critically the manuscript and final approval of the submitted version.

## Conflicts of interest

The authors declare no conflicts of interest.
